# Acute Cytomegalovirus Infection Associated With Erythema Multiforme-Like Eruption: A Case Report

**DOI:** 10.7759/cureus.54540

**Published:** 2024-02-20

**Authors:** Luca Pipitò, Antonio Cascio

**Affiliations:** 1 Infectious and Tropical Diseases Unit, Department of Health Promotion, Mother and Child Care, Internal Medicine and Medical Specialties, "G D'Alessandro, University of Palermo, AOUP P. Giaccone, Palermo, ITA

**Keywords:** immuno-competent, healthy young man, infectious mononucleosis-like syndrome, erythema multiforme-like lesions, hcmv

## Abstract

Human cytomegalovirus infection usually proceeds asymptomatically in immunocompetent patients. In symptomatic forms, mononucleosis syndrome is the most common manifestation. However, atypical cases of cytomegalovirus infections in immunocompetent subjects are reported in the literature. Here, we describe a case of cytomegalovirus-related mononucleosis syndrome that presented with an atypical erythema multiforme-like skin rash and high fever. Very few cases have been described in the literature previously. In our case, the diagnosis was supported by specific serology, and human cytomegalovirus DNA was detected in the blood sample. The clinical picture resolved without the administration of antiviral therapy.

## Introduction

Human cytomegalovirus (HCMV) belongs to the Herpesviridae family and infects most of the world's population [1]. Transmission can happen via multiple routes, including close contact, perinatal exposure, and blood transfusion [1]. Like other members of the Herpesvirus family, CMV establishes latent infection after the resolution of primary infection [1]. While the infection (primary or reactivation) can be fatal in immunocompromised patients, an asymptomatic primary infection occurs in most healthy and immunocompetent individuals. A possible manifestation of the symptomatic acute infection in healthy people is a mononucleosis-like syndrome, which can be associated with skin rash [2-4]. Here, we report an interesting case of a healthy, immunocompetent man who developed an acute cytomegalovirus infection that manifested as mononucleosis-like syndrome associated with an erythema multiforme-like eruption.

## Case presentation

**Figure 1:**
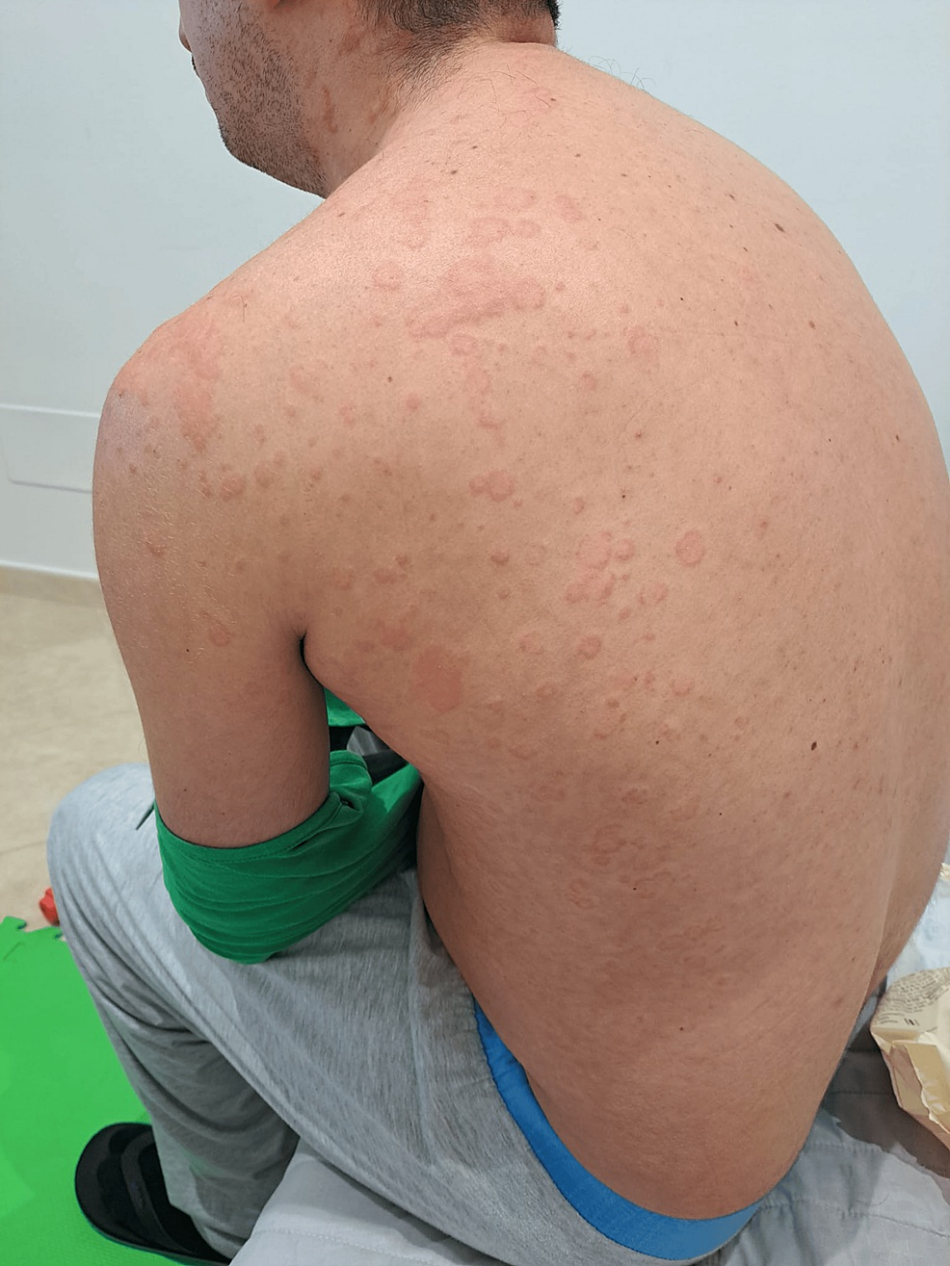
Erythematous targeted lesions on the dorsal and neck

**Figure 2:**
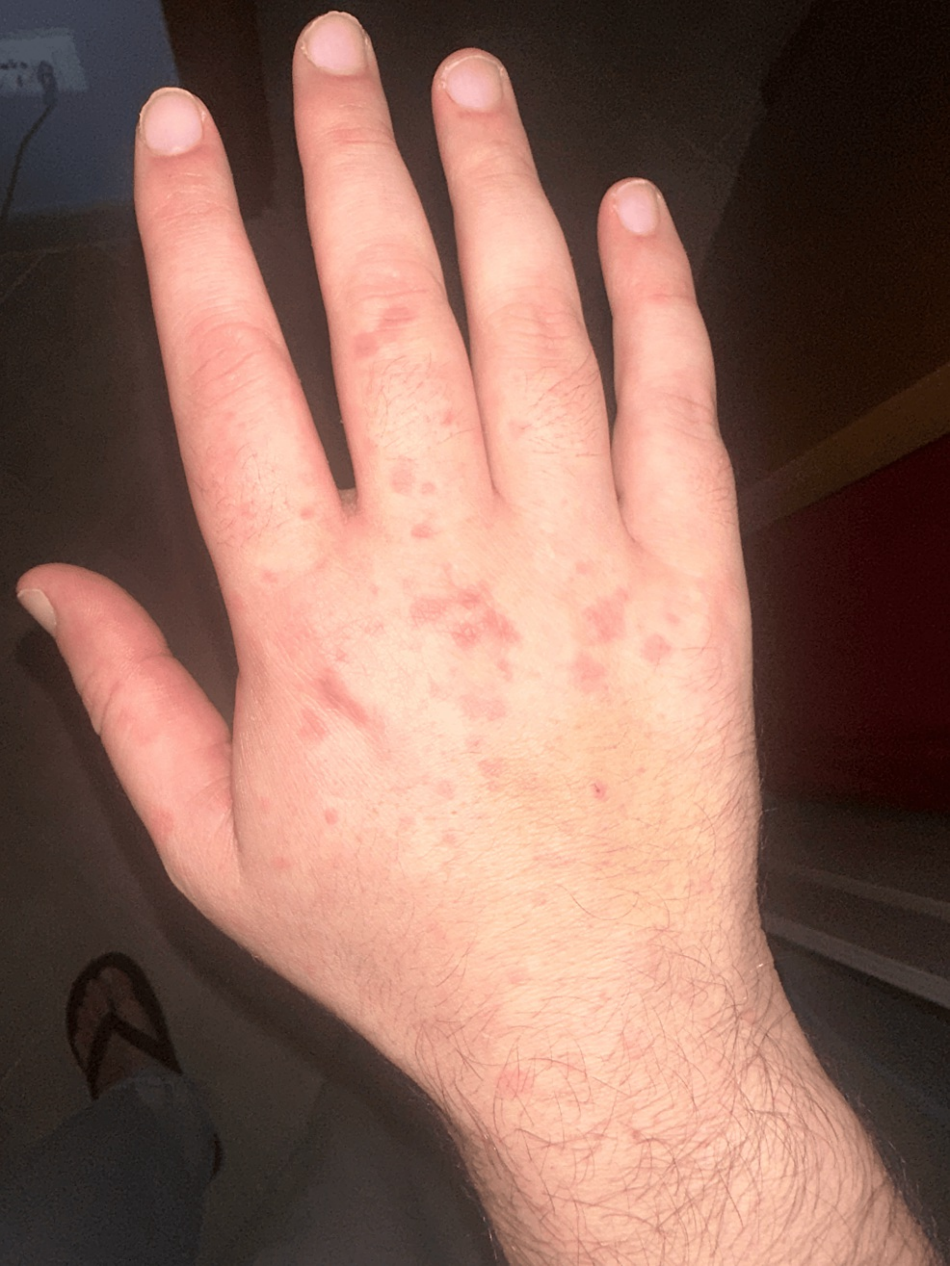
Erythematous lesions on the dorsal surface of the right hand

A previously healthy, 31-year-old man presented with a four-week history of non-itching rash and malaise. High fever (40 °C) appeared two weeks after the eruption onset. He had annular erythematous targeted lesions, some coalesced, papules on the dorsum, neck, face, and upper limbs, including hands (palms and dorsal surface) (Figure 1 and Figure 2).

He had no lymphadenopathy or mucosal or genital involvement. He denied having a sore throat, and his pharynx was normal. In the past few weeks before the presentation, the patient reported that the rash usually appeared in the morning, reduced or disappeared in the evening, and reappeared in different locations the following day. He had taken no drugs for months before the rash onset. He was in a stable relationship and denied sexual intercourse with other partners. Initially, he took doxycycline and, subsequently, prednisone without improvement. His previous medical history was unremarkable. On admission, he had a temperature of 38 °C, pulse rate of 96/min, respiratory rate of 18/min, blood pressure of 110/70 mmHg, and 98% oxygen saturation. Blood examination revealed lymphocytosis (79.6%), elevated transaminases (aspartate aminotransferase 133 U/L and alanine aminotransferase 177 U/L), mild anemia (hemoglobin 11 g/dl), and elevated C-reactive protein (33.9 mg/L) (Table 1).

**Table 1:** Blood examination

Parameters	Value	Reference
White blood cells	10270 cells/µl	4000–11000 cells/µl
Neutrophils (%)	1710 cells/µl (16.6%)	2000–8000 cells/µl (40­–74%)
Lymphocytes (%)	8170 cells/µl (79.6%)	1000-5000 cells/µl (20­–48%)
Monocytes (%)	280 cells/µl (2.7%)	160–1000 cells/µl (3­–11%)
Red blood cells	3.94 x 10^6 ^cells/µl	4.2 x 10^6 ^– 5.2 x 10^6^ cells/µl
Hemoglobin	11 g/dl	12–18 g/dl
Platelets	284 x 10^3^ / µl	150 x 10^3 ^– 450 x 10^3^/ µl
Hematocrit	32.2%	37%–52%
Aspartate aminotransferase	133 U/L	0–50 U/L
Alanine aminotransferase	177 U/L	0–50 U/L
Total bilirubin	0.51 mg/dl	< 1.2 mg/dl
Creatinine	0.99 mg/dl	0.67–1.17 mg/dl
C-reactive protein	33.9 mg/L	< 5 mg/L

Examination for human immunodeficiency virus, Hepatitis C, Hepatitis B, rickettsiosis, typhoid fever, and blood culture turned negative. Chest and abdomen computed tomography showed no pathological findings but splenomegaly (Ø 15 cm). Antinuclear antibodies showed low-level positivity (1:160), extractable nuclear antigens antibodies, and autoimmune liver disease panel resulted in negative. Epstein-Barr nuclear antigen antibodies and immunoglobulin G (IgG) anti-viral capsid antigen were positive as in a past infection. Syphilis serology was negative. A positive HCMV IgM and IgG were obtained, and blood HCMV-DNA was 4745 copies/ml. Considering the clinical, laboratory, and virological data, he was diagnosed with HCMV mononucleosis-like syndrome with erythema multiforme-like eruptions. The patient length of stay was eight days, and progressive improvement happened with the resolution first of skin eruptions and then of fever and malaise without antiviral treatment.

## Discussion

HCMV is the second most important cause of mononucleosis syndrome [2]. HCMV mononucleosis-like syndrome has been described as typhoidal in presentation because of high fever and systemic symptoms. Compared to Epstein Barr virus infectious mononucleosis, lymphadenopathies are usually absent, pharyngitis is present rarely and is mild, and adults are typically older when HCMV mononucleosis-like syndrome first manifests clinically [4,5]. However, HCMV disease can present with a wide range of manifestations, even in the immunocompetent, and pose a significant diagnostic challenge [3]. Atypical cases with gastrointestinal, hematological, cardiovascular, neurological, and urological involvement were reported [3]. As in our case, laboratory abnormalities may include lymphocytosis, elevated aminotransferases [6], mild anemia, and positive antinuclear antibodies. This last finding is common during acute or chronic infections and has no clinical relevance [7]. Although dermatologic manifestations involve one-third of patients with HCMV mononucleosis-like syndromes, showing a wide range of rashes (maculopapular, scarlatiniform, rubelliform, morbilliform, erythema, petechiae, purpura, plaques, vesicles, bullae, pustules, erosions, nodules, pustules, and vasculitis) [8], erythema multiforme (EM) was rarely reported [9,10]. EM is an acute and probably immune-mediated condition characterized by targeted lesions and possible mucosal involvement. Etiology includes infection (especially herpes simplex virus), malignancy, medications, and autoimmune diseases [11]. Koga et al. described a case of a healthy 23-year-old woman with HCMV mononucleosis-like syndrome and EM-like eruptions, characterized by erythematous annular lesions on the dorsal of the feet and on her legs, without mucosal involvement [9]. Petrosino et al. reported an EM syndrome associated with HCMV in a pediatric patient who presented with multiple erythematous target lesions and vesicles involving the face, oral cavity, hands, feet, and lower limbs [10]. Skin biopsy with immunohistochemistry with antibodies directed toward HCMV antigens may be helpful in the diagnosis of the most complex cases. In our case, a mononucleosis-like syndrome associated with EM-like eruptions without mucosal involvement was described. Serology with IgM and IgG anti-HCMV positivity were suggestive of acute infection, and blood HCMV-DNA confirmed the diagnosis. IgG antibodies were positive because the onset of the symptoms occurred four weeks before the patient's admission. Thus, considering the resolution of the clinical manifestations and the virology findings, a skin biopsy was considered unnecessary. To the best of our knowledge, our case is unique due to the fleeting nature of the rash, which appeared in the morning and reduced or disappeared in the evening. No other similar cases were reported in the literature.

## Conclusions

HCMV mononucleosis-like syndrome should be considered during the differential diagnosis of patients with skin lesions of an unknown nature and fever. Cutaneous manifestations may be heterogeneous, and HCMV may be associated with erythema multiforme-like eruption. HCMV serology and viral DNA testing are two essential tools to confirm the diagnosis. A skin biopsy may not be necessary.
